# Genome-wide identification and expression analysis of *ARF* gene family in embryonic development of Korean pine (*Pinus koraiensis*)

**DOI:** 10.1186/s12870-024-04827-w

**Published:** 2024-04-10

**Authors:** Yue Zhang, Wei Wu, Hailong Shen, Ling Yang

**Affiliations:** 1 State Key Laboratory of Tree Genetics and Breeding, College of Forestry, Northeast Forestry University, Harbin, 150040 China; 2 State Forestry and Grassland Administration Engineering Technology Research Center of Korean Pine, Harbin, 150040 China

**Keywords:** Korean pine (*Pinus koraiensis*), Auxin response factor, Gene family, Phylogenetic analysis, Expression analysis, Embryonic development

## Abstract

**Background:**

The Auxin Responsive Factor (*ARF*) family plays a crucial role in mediating auxin signal transduction and is vital for plant growth and development. However, the function of *ARF* genes in Korean pine (*Pinus koraiensis*), a conifer species of significant economic value, remains unclear.

**Results:**

This study utilized the whole genome of Korean pine to conduct bioinformatics analysis, resulting in the identification of 13 *ARF* genes. A phylogenetic analysis revealed that these 13 *PkorARF* genes can be classified into 4 subfamilies, indicating the presence of conserved structural characteristics within each subfamily. Protein interaction prediction indicated that Pkor01G00962.1 and Pkor07G00704.1 may have a significant role in regulating plant growth and development as core components of the PkorARFs family. Additionally, the analysis of RNA-seq and RT-qPCR expression patterns suggested that *PkorARF* genes play a crucial role in the development process of Korean pine.

**Conclusion:**

*Pkor01G00962.1* and *Pkor07G00704.1*, which are core genes of the PkorARFs family, play a potentially crucial role in regulating the fertilization and developmental process of Korean pine. This study provides a valuable reference for investigating the molecular mechanism of embryonic development in Korean pine and establishes a foundation for cultivating high-quality Korean pine.

**Supplementary Information:**

The online version contains supplementary material available at 10.1186/s12870-024-04827-w.

## Background

Auxin plays a crucial role in numerous processes related to plant growth and development. These processes include regulating the cell cycle, facilitating embryo and organ development, promoting adventitious root formation, and aiding in fruit and seed development. Additionally, auxin also exhibits responsiveness to external environmental stimuli [1, 2]. Auxin primarily exists in tissues with a high rate of cell division and is transported between different tissues and cells through carrier proteins [3]. Treatment with exogenous auxin at varying concentrations can impact the transcription of the auxin response factor *ARF*, consequently regulating the initiation of seed development signals [4]. Most ARF proteins are composed of three conserved domains, the B3 DNA binding domain (DBD) in the N-terminal region, the C-terminal dimerization domain (CTD) that controls protein-protein interactions, and the variable middle region (MR) [5]. When the auxin concentration in cells is low, the (Aux/IAA) protein and the ARF protein form homo- or heterodimers (ARF-ARF, ARF-Aux/IAA) in the C-terminal region, which inhibits the activity of the ARF protein [5–7]. When the auxin concentration is high, the Aux/IAA repressor protein is degraded through the ubiquitin-proteasome pathway, thereby releasing ARF to activate the expression of downstream auxin-responsive genes [8, 9].

With the advancement in genome sequencing, *ARF* genes have been identified in various species, including *Arabidopsis* (*Arabidopsis thaliana*) [10], rice (*Oryza sativa*) [11], Populus (*Populus trichocarpa*) [12], Soybean (*Glycine max*) [13], maize (*Zea mays*) [14]. The first *ARF* gene, *AtARF1*, was initially discovered in the model plant *Arabidopsis* [10]. In the *Arabidopsis* genome, there are a total of 23 members of the *ARF* gene family. Mutations in the *ARF* gene have been found to cause alterations in various biological functions [10]. Mutants of *atarf1* and *atarf2* display abnormal stem and leaf morphology, as well as delayed flowering [15, 16]. T-DNA insertion mutants of *atarf3* exhibit an increased number of sepals [17]. Mutants of *atarf5* are incapable of developing normal cotyledon and root tissues [18]. *atarf7* is associated with photomorphogenesis and regulates plant phototropism in response to blue light [19]. *AtARF8* plays a role in hypocotyl and stem growth, as well as in the regulation of fertilization and fruit development [20, 21]. In addition, it has been discovered that *AtARF7*, *AtARF8*, and *AtARF19* play a role in transmitting the auxin signal in the root [21, 22]. Recent studies have shown that post-translational modifications are primarily involved in auxin signaling in root structures. Specifically, SUMOylation has been found to negatively regulate the DNA-binding activity of ARF7, which consequently impacts the expression of auxin-responsive genes associated with lateral root formation [23]. SUMOylation plays a crucial role in enhancing the stability of MdARF8 protein, thereby facilitating the development of apple lateral roots [24]. Auxin can regulate anthocyanin biosynthesis through the Aux/IAA-ARF signaling pathway. MdARF13 interacts with MdIAA121 to regulate anthocyanin biosynthesis during apple fruit ripening [25]. In addition, EkARF5.1 directly interacts with auxin response elements to regulate anthocyanin synthesis during fruit ripening in Euscaphis konishii [26]. In the case of Populus, the nuclear interaction between PtrARF23 and PtrIAA10, as well as PtrIAA28, is responsible for regulating wood formation [12]. The peu-miR160a-PeARF17.1/PeARF17.2 module is an important regulator involved in the development of the adventitious roots of poplar [27]. In addition to regulating plant growth and development, ARF proteins have been found to play a crucial role in abiotic stress responses in different plant species. For instance, the expression levels of *ARF1*, *ARF4*, *ARF6B, ARF10A*, and *ARF18* genes were observed to significantly increase in tomato leaves subjected to drought stress [28]. The genes *OsARF11* and *OsARF15* in rice, as well as *AcARF4*, *AcARF5*, *AcARF23a*, and *AcARF28a* in kiwifruit (*Actinidia*), were found to be significantly up-regulated after salt stress induction [29, 30]. While the diversity and complexity of the *ARF* gene family in different species have been extensively studied, there is still a lack of research on the function of *ARF* genes in Korean pine. Korean pine is a type of high-quality wood that produces pine nuts containing rich vitamins, proteins, and other essential nutrients. Due to its high economic, ecological, and edible value, further investigation into the *ARF* genes in Korean pine is warranted. Therefore, based on the molecular functions of *ARF* in embryonic development, seed germination, growth and development, stress response, and fruit ripening in different species, we hypothesized that the identification and functional resolution of *ARF* gene members in Korean pine would be a guiding value for the cultivation of high-yielding and high-quality Korean pine varieties [31–34]. Exploring the biological functions of *ARF* genes at different developmental stages is of great significance for the large-scale production of Korean pine resources. This study aimed to identify and characterize 13 *PkorARF* genes based on the whole genome of Korean pine. The research focused on determining their chromosomal location, gene structure, and phylogenetic relationships with other species. By constructing the protein interaction network, it was discovered that two members of PkorARF interact with the core protein. Furthermore, the cis-acting regulatory elements and dynamic expression patterns of *ARF* at different developmental stages suggest that *ARF* plays a crucial role in the growth and development process. These findings provide valuable insights for the optimization of Korean pine germplasm resources.

## Methods

### Identification of the *ARF* gene family in Korean pine

The complete genome and annotation information of Korean pine were obtained from our laboratory. To identify the sequence of the *ARF* gene in Korean pine, we compared the BLAST results using ClustalW2.1 [35], Pfam (http://pfam.xfam.org/) [36], and NCBI CD search programs (https://www.ncbi.nlm.nih.gov/Structure/cdd/wrpsb.cgi) [37] to eliminate redundant sequences and identify members of the *PkorARF* family. The amino acid and CDS length information of the *PkorARF* genes was downloaded from the Ensembl plants (https://plants.ensembl.org/) [38]. Bioinformatics analysis was conducted on the *PkorARF* gene using various tools. ExPASy (http://www.expasy.ch/tools/pi_tool.html) [39] was utilized to determine the molecular weight (MW) and isoelectric point (pI) of PkorARFs. TMHMM (http://www.cbs.dtu.dk/services/TMHMM) [40] was employed to predict the transmembrane domain of the PkorARF protein. Additionally, the Plant-Ploc website (http://www.csbio.sjtu.edu.cn/) [41] was used to predict the subcellular localization, hydrophobicity, and stability. Finally, the protein tertiary structure was analyzed using the SWISS MODEL (https://swissmodel.expasy.org/) homology modeling method [42].

### Analysis of *PkorARF* gene structure and protein structure

To analyze the structure of each gene, we compared the CDS of the *PkorARF* gene family with its corresponding genomic DNA sequence. The gene exon-intron structure diagram was then mapped using the Gene Structure View (Advanced) feature of TBtools [43]. Protein conserved motifs were predicted using the MEME online tool (https://meme-suite.org/) [44] with a motif value set to 20. These motifs were subsequently mapped using TBtools [43]. Finally, we combined the Gene Structure, protein conserved motifs, and protein domains based on the Gene Structure View (Advanced) function of TBtools [43].

### Sequence alignment and phylogenetic analysis of *PkorARF* family genes

ARF protein sequences from *Arabidopsis* were obtained from TAIR (https://www.arabidopsis.org/), while ARF protein sequences from rice were downloaded from Phytozome (https://phytozome.jgi.doe.gov/pz/). The ARF protein sequences in Populus were obtained from a study by Kalluri et al. [45], Phylogenetic trees were constructed with MEGA 7.0 [46] using the neighbour-joining method and visualised using the iTOL (https://itol.embl.de/) [47].

### Analysis of cis-acting elements of the *PkorARF* gene promoters

The 2000 bp sequence upstream of the *PkorARF* genes was selected to identify cis-elements in the promoter region, based on the identified *ARF* gene family in Korean pine. PlantCARE (http://www.dna.affrc.go.jp/PLACE/) [48] was used in this study to predict the cis-regulatory elements present in the promoter sequence. Cis-acting elements with the same function were uniformly named. The analysis results were visualized using the Simple BioSequence Viewer function in TBtools [43].

### Protein interaction and GO enrichment analysis

The interactions between PkorARFs were analyzed using the STRING website (https://cn.string-db.org/) [49]. Protein sequence analysis of PkorARFs was performed with a confidence level of 0.4, and the output result was visualized using Cytoscape [50]. For GO enrichment analysis, the biological processes, cellular components, and molecular functions of the 13 *PkorARF* genes were analyzed using STRING (https://cn.string-db.org/). The results were visualized using microscopic letter website (http://www.bioinformatics.com.cn/).

### Gene expression profiles of PkorARFs based on RNA-seq

RNA-seq data of *PkorARF* genes in three developmental periods of Korean pine were obtained from our previous study [51]. The Korean pine ovules were obtained from the Hongwei seed garden, Baishan City, Jilin Province, China (127°27′32″ E, 42°13′36″ N), family 425. The study was performed on elite clonal grafted mother trees colonized in 1989. The collection was performed in June of the second year (2021) after natural pollination. The samples for the one week before fertilization were obtained on June 15 (ZB). The samples for the week of fertilization were obtained on June 22 (ZC), and the samples for the one week after fertilization were obtained on June 30 (ZD). RNA-seq data are deposited on the NCBI website (Bioproject ID: SUB12731425). The gene expression data was log2 transformed based on Fragments Per Kilobase of transcript per Million fragments mapped (FPKM) and utilized for cluster analysis. Gene expression heatmaps were visualized using the microscopic letter website (http://www.bioinformatics.com.cn/).

### RNA extraction and RT-qPCR analysis

The stages of Korean pine include ovule development one week prior to fertilization (ZB), ovule fertilization (ZC) and ovule development one week after fertilization (ZD). The total RNA of ZB, ZC, and ZD was extracted using the Omega Bio-Tek EZNA plant RNA extraction kit. Reverse transcription amplification was carried out using the Goldenstar RT6 cDNA Synthesis Mix. Subsequently, amplification was performed using the ABI 7500 Rapid Real-Time Fluorescence Quantitative PCR System and the 2×T5 Fast qPCR Mix (SYBR Green I). The internal reference genes, 18 S and β-actin, were used and the relative expression abundance was calculated using the 2^−ΔΔCt^ method. The primer sequences can be found in Supplementary Table 1.

### Statistical analysis

Statistical analyses were conducted using SPSS 13.0 software (https://www.ibm.com/analytics/spss-statistics-software). Each group consisted of three biological replicates. Significant differences were assessed using one-way analysis of variance (ANOVA) and displayed as *P* < 0.05.

## Results

### Identification and characterization of the *PkorARF* genes

**Fig. 1:**
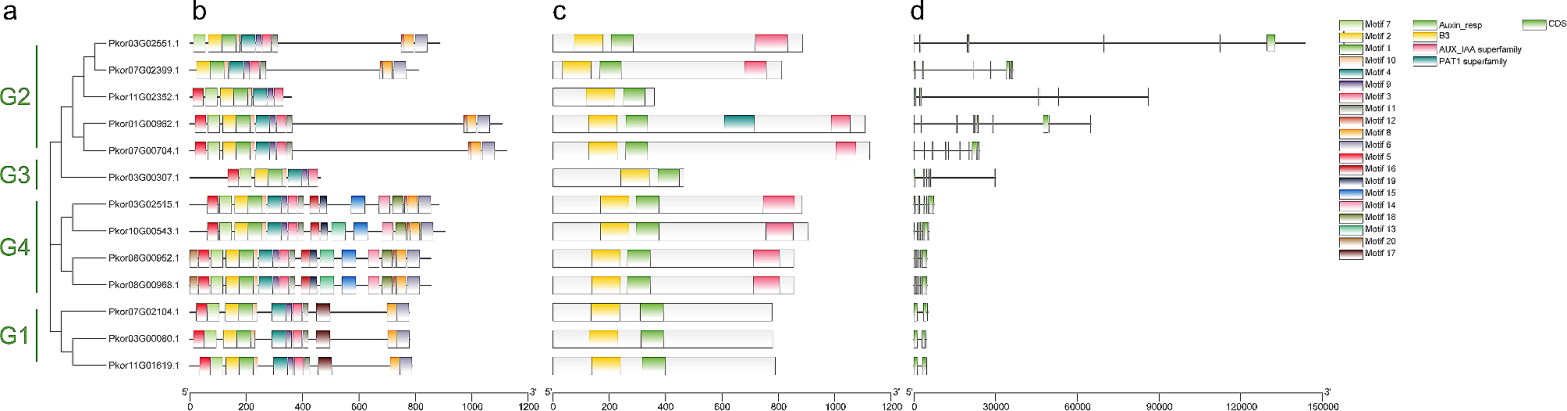
Gene and protein structure of 13 PkorARFs. Phylogenetic relationships of the 13 *PkorARF* genes (**a**), Protein motifs (**b**), Protein conserved structural domains (**c**), Gene structure (**d**). G1-G4 represent PkorARFs distributed in different subfamilies, and the horizontal coordinate represents the length of the gene/amino acid sequence

To identify members of the *ARF* gene family in Korean pine, we initially aligned the known ARF protein sequences of *Arabidopsis* with the Korean pine protein database. This step allowed us to identify potential *PkorARF* genes. To further validate our findings, we utilized the Pfam database (http://pfam.xfam.org) and the CDD Search program (https://www.ncbi.nlm.nih.gov/Structure/cdd/wrpsb.cgi) to confirm the presence of conserved domains in these ARF proteins. After removing the unqualified sequences, 13 *PkorARF* genes were finally identified and named *PkorARF1* to *PkorARF13* according to their chromosomal locations (Supplementary Table 2). All of these genes contain complete B3 DNA-binding domain (DBD) and Auxin responsive domains (ARF) (Fig. 1). Among them, Pkor11G02352.1, Pkor03G00307.1, Pkor07G02104.1, Pkor03G00080.1, and Pkor11G01619.1 lack C-terminal AUX/IAA domains (CTD), while the other members of PkorARF all have typical ARF domains (DBD-ARF-CTD) (Fig. 1). Intron/exon structure analysis of *PkorARF* genes can provide valuable insights into the evolutionary relationships between species [52]. The exon and intron structures of *PkorARF* genes were identified using the Gene Structure Display Server (GSDS) online tool. Figure 1 illustrates that the structures of different genes within the same subfamily exhibit both conserved regions and differential regions. To identify conserved motifs in PkorARF proteins, the MEME program (https://meme-suite.org/meme/tools/meme) was utilized. A total of 20 motifs with variable amino acid length were identified in 13 PkorARFs, including motif1-motif20. The G1 subfamily exhibits 12 conserved motifs, the G2 subfamily has 11 conserved motifs, the G4 subfamily has 19 conserved motifs, and the G3 subfamily has 8 conserved motifs. Among these four subfamily members, there are 6 common motifs, namely motif1-4, motif9, and motif10, suggesting that these subfamily members may have similar functions. Furthermore, the majority of PkorARF proteins contain motif 2 in DBD and motif 3 in CTD. Most PkorARFs follow the same motif order, which is motif 2, motif 1, motif 10, motif 4, motif 9, and motif 3 (Fig. 1).

Detailed characteristics of PkorARFs, including gene and protein sequence length, location, and physicochemical parameters, are presented in Supplementary Tables 2 and Supplementary Table 3. The length of the coding sequences (CDS) ranged from 1083 to 3375 bp, and the corresponding protein sequences ranged from 360 to 1124 amino acids (aa) (Supplementary Table 4). The molecular weights (MW) varied from 40.29 to 126.10 kDa, while the theoretical isoelectric points (pI) ranged from 5.76 to 9.16. Analysis using TMHMM Server v2.0 revealed that none of the PkorARF proteins possess transmembrane structural domains. Furthermore, subcellular localization and protein stability predictions indicated that all 13 PkorARF proteins were located in the nucleus and exhibited instability. The hydrophobicity of the 13 PkorARF proteins was found to be less than 0, classifying them as hydrophilic nuclear proteins (Supplementary Table 2).

### Phylogenetic analysis of PkorARFs

**Fig. 2:**
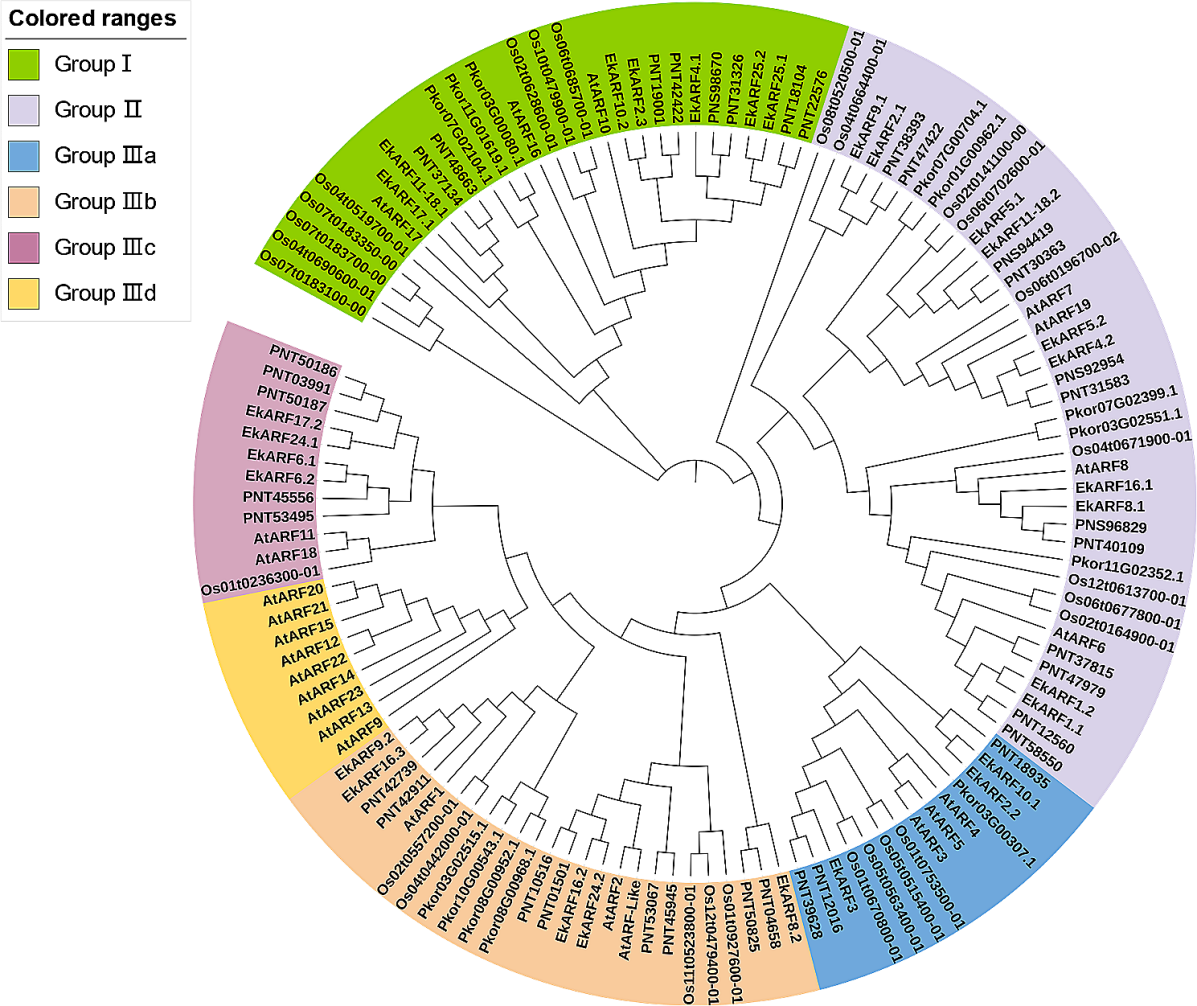
Phylogenetic relationship of ARF from Korean pine, Arabidopsis, rice, *Euscaphis konishii* and Populus. Phylogenetic trees were constructed using MEGA 7.0 and using the Neighbour-Joining (NJ) method with 1000 duplicates. All ARF genes were divided into three Groups with different colours. GroupI-GroupIII on the left side indicate several ARF family members of Korean pine, Arabidopsis, rice, *Euscaphis konishii* and Populus distributed in six different subfamilies. The branches of each subfamily are indicated by specific colour and different members of the same subfamily have the same color

Phylogenetic analysis was conducted to examine the relationships between ARFs among different species and explore the potential functions of PkorARFs. A phylogenetic tree was constructed using full-length ARF protein sequences from *Arabidopsis*, rice, *Euscaphis konishii* and Populus (Fig. 2). The analysis revealed that the *ARF* gene of Korean pine shared similarities with *Arabidopsis*, rice, *Euscaphis konishii* and Populus, and all of them were classified into 3 subfamilies. Group I and Group II consisted of 3 and 5 *PkorARF* genes, respectively, with Group II having the highest number of *ARF* genes in Korean pine and Populus. Group III comprised four subclasses. Group IIIa contained only one *PkorARF* gene (*Pkor03G00307.1*), while Group IIIb consisted of four *PkorARF* genes (*Pkor08G00952.1*, *Pkor08G00968.1*, *Pkor10G00543.1*, and *Pkor03G02515.1*). The Group IIIb subfamily had the fewest *AtARF* gene members, and *Pkor08G00952.1*, *Pkor08G00968.1*, *Pkor10G00543.1*, and *Pkor03G02515.1* exhibited the closest genetic relationship with *AtARF1* and *AtARF2* (Fig. 2). Group IIIc and Group IIId did not contain *PkorARF* genes, with the Group IIId subfamily exclusively consisting of *AtARF* members in *Arabidopsis* (Fig. 2).

### Chromosomal location of *PkorARF* genes

**Fig. 3:**
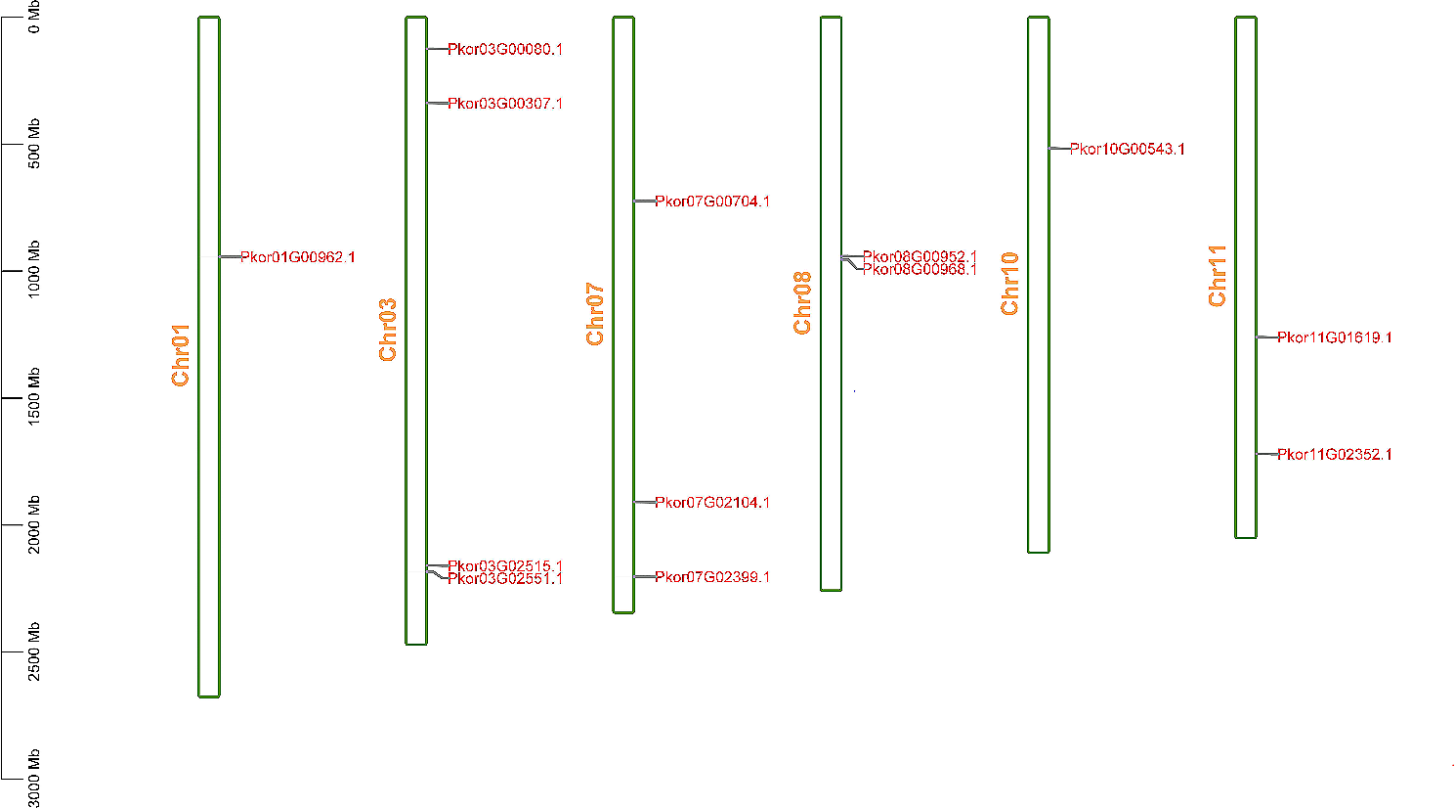
Chromosomal distribution of *PkorARF* genes in Korean pine. The vertical coordinate indicates the chromosome length, the green bar indicates the chromosomes of Korean pine, and the red color represents all *PkorARF* genes, each of which maps onto a chromosome

To investigate the chromosomal locations of *PkorARF* genes, we analyzed the distribution of these genes on Korean pine chromosomes (Fig. 3). We found that a total of 13 *PkorARF* genes were mapped unevenly across the chromosomes. Notably, chromosome 3 exhibited the highest number of PkorARFs, while chromosome 1 had the fewest. Chromosome 7 contained three PkorARF genes, whereas chromosomes 8 and 11 each harbored two *PkorARF* genes (Fig. 3).

### Protein structure and protein interaction prediction of PkorARFs in Korean pine

**Fig. 4:**
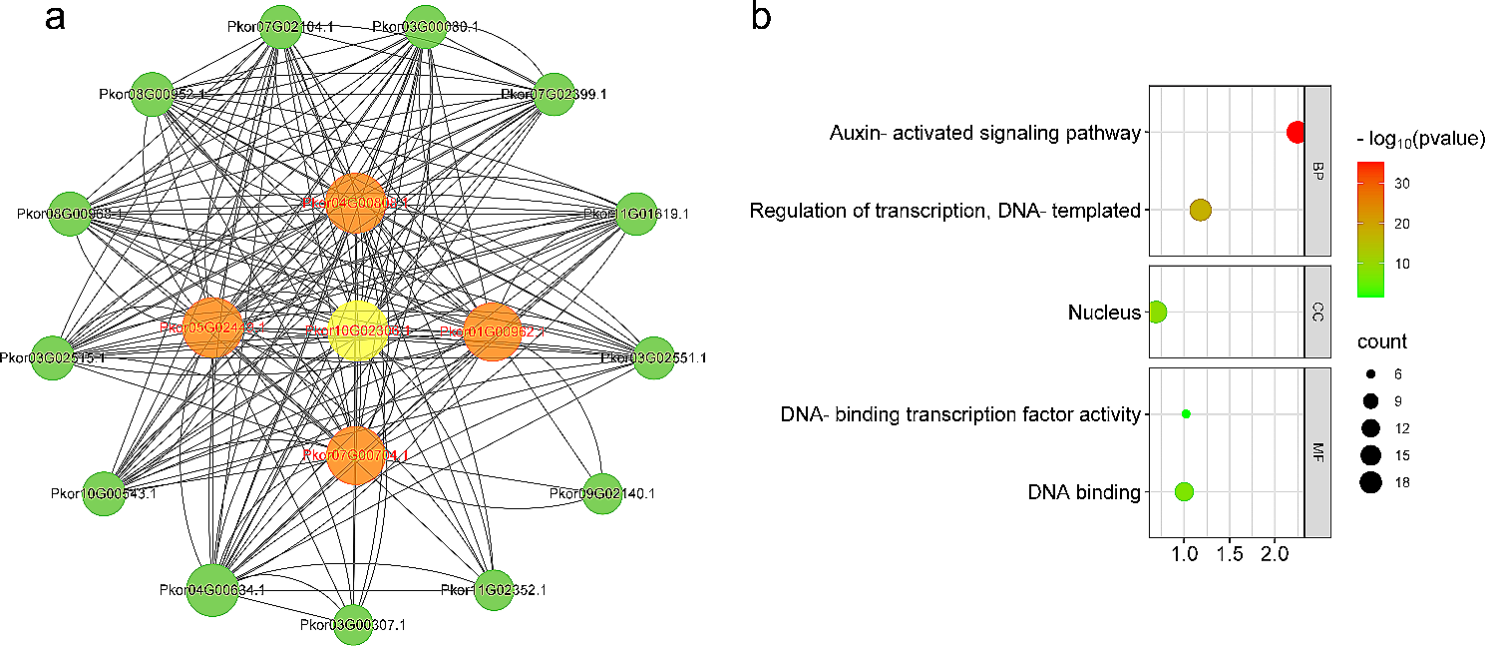
Protein interaction network (**a**) and GO enrichment analysis (**b**) between *PkorARF* genes. The internal yellow and orange circles represent the core members of PkorARFs in Fig. 4A, while the external green circles indicate other members of PkorARFs. Gray lines represent the interaction relationship between all members of the PkorARFs gene family. The horizontal scale indicates the ratio of the number of genes in the pathway to the number of *PkorARF* gene family members, and the vertical scale indicates the functions of PkorARFs, including cellular components, molecular functions, and biological processes in Fig. 4b. The color of the circle indicates the significance of the proportion of genes in the pathway to the total genes (*P* < 0.05 is considered significant). The size of the black circle indicates the number of *PkorARF* genes in the pathway

To determine the protein structure of PkorARF, we used the SWISS-MODEL (https://swissmodel.expasy.org/) to predict homology models. The results showed successful modeling for all PkorARFs (Supplementary Fig. 1). To analyze potential interactions between PkorARFs, we utilized STRING (https://cn.string-db.org/) and compared the protein sequences of all PkorARFs in Korean pine to identify the highest scoring homologous proteins. The results were visualized using Cytoscape. The comparison of PkorARFs protein sequences with the STRING database revealed an 18-node protein interaction network with 42 edges (Fig. 4). Among the PkorARFs, 8 members were located on the outside of the protein-interaction network, while Pkor01G00962.1 and Pkor07G00704.1 were positioned inside the network, suggesting that they may be the core components of the *PkorARF* gene family in regulating plant growth and development. Notably, Pkor10G02306.1 occupied a central position and exhibited potential interactions with all PkorARF proteins in the network. Furthermore, GO enrichment results indicated that PkorARFs are primarily involved in biological processes such as the auxin-activated signaling pathway and regulation of transcription (Supplementary Table 5). These PkorARFs are mainly located in the nucleus and may function as transcription factors (Fig. 4).

### Cis-acting elements of *PkorARF* genes promoter

**Fig. 5:**
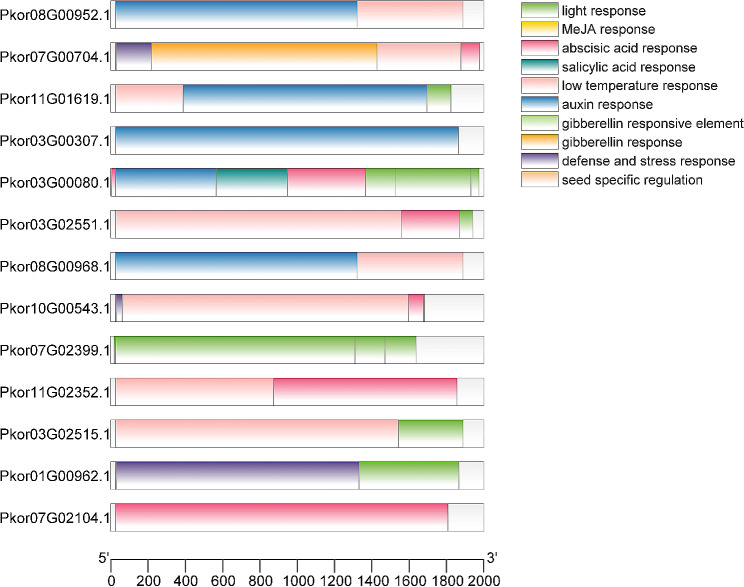
Cis-acting element analysis of *PkorARF* genes promoter. 13 *PkorARF* genes were classified according to their phylogenetic relationship. The horizontal coordinates represent gene lengths and the vertical coordinates represent members of PkorARFs. Different colours represent light, MeJA, abscisic acid, salicylic acid, auxin, gibberellin, stress response and seed development regulatory elements

Cis-acting elements within the promoter region of *PkorARF* genes were analyzed to investigate their biological functions. The analysis revealed that, apart from the core promoter regions like TATA-box and CAAT, the cis-acting elements in the *PkorARF* genes promoter region mainly consisted of light response, hormone response, stress response, and seed development regulatory elements (Fig. 5). Plant hormone response elements constituted the largest proportion, including auxin response elements (TGA, AuxRR-core), ABA response elements (ABRE), gibberellin response elements (P-box, GARE motif, TATC-box), MeJA response elements (CGTCA-motif, TGACG-motif), and salicylic acid-responsive element (TCA) (Supplementary Table 6). Additionally, the promoter region of the *PkorARF* genes contained various stress-responsive elements, such as the drought-responsive element MBS with a MYB binding site and the element LTR related to low temperature response (Fig. 5).

### Expression patterns of *PkorARF* genes at different developmental stages

**Fig. 6:**
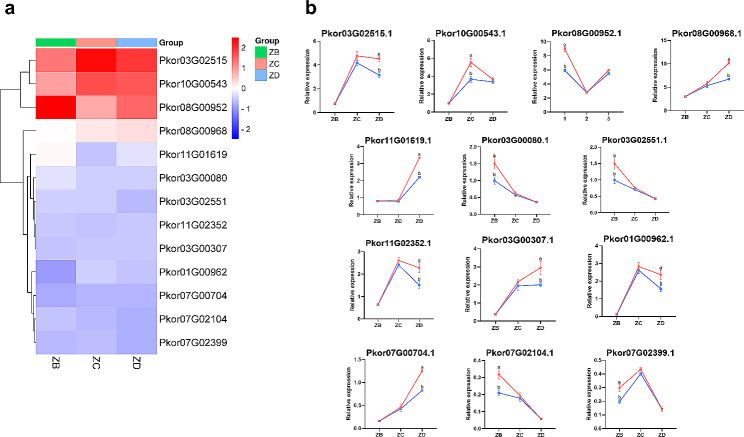
RNA-seq of *PkorARF* genes at different developmental stages (ZB, ZC and ZD ) (**a**), RT-qPCR expression at different developmental stages (**b**). Based on RNA-seq results, we analyzed the expression patterns of all *PkorARF* genes at three embryonic developmental stages (ZB, ZC and ZD). Korean pine housekeeping genes 18 S and β-actin were used as internal references, and the expression of *Pkor10G00543.1* in ZB was used as a control. The red and blue folded lines represent two internal reference genes, respectively, and the horizontal coordinates represent three embryonic developmental stages of Korean pine. The vertical coordinates represent the relative expression levels of *PkorARF* genes, and gene names are labeled at the top of the graph

To investigate the functions of ARF in plant development, we analyzed the expression of PkorARFs at different developmental stages using RNA-seq data of ZB, ZC, and ZD in Korean pine (Supplementary Table 7). The results indicate that *Pkor03G02515.1*, *Pkor10G00543.1*, *Pkor08G00952.1*, and *Pkor08G00968.1* from the Group IIIb subfamily consistently exhibited high expression levels during all stages of embryonic development in Korean pine. In contrast, the other members of PkorARFs displayed relatively low expression levels during embryonic development. The genes *Pkor03G02515.1*, *Pkor10G00543.1*, and *Pkor08G00968.1* showed high expression levels during the ZC and ZD stages (Fig. 6). On the other hand, *Pkor08G00952.1* exhibited the highest expression in ZB (Fig. 6). While the expression level of *Pkor11G01619.1* was highest in ZB, it was significantly reduced in ZC and ZD (Fig. 6), indicating its predominant role in early embryonic development rather than late embryonic development. Furthermore, the expression levels of *Pkor01G00962.1* and *Pkor07G00704.1* were lowest in ZB, but significantly increased in ZC and ZD (Fig. 6), implying that *Pkor01G00962.1* is primarily involved in late embryonic development.

To ensure the reliability of the RNA-seq data, we conducted additional analysis on the expression pattern of *PkorARF* genes during the development of Korean pine embryo using RT-qPCR. The expression patterns of these *PkorARF* genes in embryos at different developmental stages were found to be highly consistent with the RNA-seq results, as depicted in Fig. 6. The expression patterns of *PkorARF* genes were categorized into five groups. Specifically, *Pkor03G00307.1*, *Pkor07G00704.1*, and *Pkor08G00968.1* exhibited up-regulation throughout embryonic development. On the other hand, *Pkor01G00962.1*, *Pkor03G02515.1*, *Pkor07G02399.1*, and *Pkor10G00543.1* showed a trend of up-regulation from ZB to ZC and down-regulation from ZC to ZD. *Pkor08G00952.1* and *Pkor11G01619.1* displayed a down-regulation from ZB to ZC and an increase from ZC to ZD. Both *Pkor03G00080.1* and *Pkor07G02104.1* exhibited a consistent down-regulated expression throughout embryonic development.

## Discussion

### Identification and structural analysis of *PkorARF* genes in Korean pine

Auxin, one of the earliest discovered major plant hormones, plays a crucial role in regulating the growth and development of plants [53]. *ARF* transcription factors are key genes in auxin response and mediate the auxin signal transduction pathway [54]. The *ARF* gene was initially identified to have 23 members in the model plant *Arabidopsis* and has since been identified in multiple species [5]. Rice has 25 *ARF* members [11], apple (*Malus domestica*) has 31 *ARF* members [55], silver birch (*Betula pendula*) has 17 members [56], birch (*Betula platyphylla*) has 15 members [57], *Euscaphis konishii* has 29 members [26], and Populus has 35 members [12]. However, the *ARF* gene has not yet been identified in Korean pine. In this study, we conducted a bioinformatics analysis of the whole genome of Korean pine and identified a total of 13 *PkorARF* genes. These genes were found to be unevenly distributed on six chromosomes of Korean pine, with chromosome 1 being the longest but having the least number of genes. Similarly, in both Populus and *Populus yunnanensis*, which have 19 chromosomes, the *ARF* genes were distributed on 16 chromosomes, and the distribution of genes on different chromosomes was also uneven. Chromosome 1 was found to be the longest in both Populus and *Populus yunnanensis*, but it had fewer *ARF* genes [12, 58]. The analysis indicated that *ARF* genes are distributed similarly on the chromosomes of woody plants, and the number of *ARF* genes on each chromosome is independent of chromosome length. Most members of PkorARFs contain the typical DBD-ARF-CTD domains. However, Pkor11G02352.1, Pkor03G00307.1, Pkor07G02104.1, Pkor03G00080.1, and Pkor11G01619.1 lack the CTD domain (Fig. 1). CTDs control protein-protein interactions and can form dimers with ARF structural domains, which, in turn, regulate the transcriptional activity of genes [10, 11]. However, the five PkorARF members lacked the CTD structural domain, which resulted in their inability to form dimers with other *ARF* members for regulating the expression of auxin-responsive genes. The proportion of PkorARF members lacking CTD domains in Korean pine (38.5%) is higher than that in other species, such as *Arabidopsis* (17.39%) [10], tomato (*Solanum lycopersicum*) (28.6%) [59], *Brassica juncea var. tumida* (22.6%) [60], and Populus (37.5%) [12]. However, the proportion of structural domains lacking in PkorARF (38.5%) is similar to that of PtrARF (37.5%), suggesting both variability in structure among different species and similarity in structure among closely related species.

### Phylogenetic relationship of *ARF* gene family members in Korean pine

The function of the *ARF* gene has been extensively studied in various species, providing a reference for exploring the potential functions of *PkorARF* gene family members in Korean pine [61]. To determine the biological function of the *PkorARF* genes, we constructed a phylogenetic tree comparing Korean pine with different species (Fig. 2). The results revealed that all ARF proteins were classified into 6 subfamilies, with Group IIId consisting solely of AtARFs. This finding is consistent with previous studies on *ARF* genes in poplar [62], and eggplant (*Solanum melongena*) [63], suggesting significant changes in the functions of *AtARF* family members in Group IIId during species evolution. Group II contained the highest number of *ARF* genes from Korean pine and Populus (Fig. 2). This suggests that *PkorARF* clusters have a preference for the same subfamily as *PtrARF* and have a closer evolutionary relationship compared to *AtARF* and *OsARF*. In *Arabidopsis*, *AtARF1* and *AtARF2* demonstrate functional redundancy and play a role in promoting floral organ abscission and fruit development [15]. *CsARF2-1* and *CsARF2-2* in tea plant, as well as *AtARF2*, all belong to the same subfamily and show high expression levels in floral organs [64]. In Group IIIb, *Pkor08G00952.1*, *Pkor08G00968.1*, *Pkor10G00543.1*, and *Pkor03G02515.1* exhibit the closest genetic relationship with *AtARF1* and *AtARF2* (Fig. 2). This suggests that *Pkor08G00952.1*, *Pkor08G00968.1*, *Pkor10G00543.1*, and *Pkor03G02515.1* may play a role in regulating the development of the reproductive organs in Korean pine. *AtARF3* has been shown to potentially inhibit cytokinin biosynthesis during organ development, thereby regulating meristem activity [65]. As a member of the same Group IIIb subfamily as *AtARF3*/*4*/*5*, *Pkor03G00307.1* may also play a role in organ development. Additionally, *AtARF7* and *AtARF19* have been identified as regulators of plant embryo development, root growth, and seedling growth [66]. *Pkor07G00704.1*, *Pkor01G00962.1*, *Pkor07G02399.1*, *Pkor03G02551.1*, and *Pkor11G02352.1* exhibited the highest genetic similarity with *AtARF7* and *AtARF19* in the Group II subfamily (Fig. 2). This indicates that *AtARF7* and *AtARF19* could potentially play a role in regulating embryonic developmental processes in Korean pine.

### Protein interaction network analysis and cis-acting element analysis were conducted on PkorARFs

Protein blast results indicated that *Pkor10G02306.1* exhibited significant homology with *IAA8*, *IAA14*, and *IAA12*, which are members of the *Aux/IAA* gene family. Similarly, *Pkor01G00962.1* and *Pkor07G00704.1* showed high homology with *AtARF5*, *AtARF7*, and *AtARF19*. Moreover, *Pkor01G00962.1* and *Pkor07G00704.1* are part of the same subfamily as *AtARF6*/*7*/*8*/*19*, suggesting that they might share similar biological functions (Fig. 2). As a typical transcription factor, *ARF* can be regulated by upstream genes and regulate the expression of downstream genes. *Aux*/*IAA*, an early auxin response factor, has been confirmed to interact with *ARF* in mediating auxin signaling [67]. . *Arabidopsis* has a total of 29 *Aux*/*IAA* genes, among which *IAA14* plays a role as a negative regulator for *ARF7* and *ARF19*. This regulation occurs through the interaction between *IAA14* and *ARF7*/*ARF19*, ultimately influencing the rate of lateral root development [68]. Another gene, *IAA8*, interacts with the downstream factor *ARF6*/*8*, leading to changes in floral organ development in *Arabidopsis*. This interaction is mediated by the modulation of jasmonic acid (JA) levels. *IAA8* interacts with the downstream factor *ARF6*/*8*, influencing floral organ development in *Arabidopsis* by modulating the levels of jasmonic acid (JA) [69]. In addition, *IAA12* plays a role in the differentiation of the meristem during early embryonic development of *Arabidopsis* through its interaction with *ARF5* [70]. The results indicate that *Pkor10G02306.1* may have an interaction with *Pkor01G00962.1* and *Pkor07G00704.1*, which are members of *PkorARF*, in order to regulate the development of lateral roots, floral organs, and early embryonic meristem.

The homologous sequence blast results revealed a high degree of similarity between *Arabidopsis IAA16* and *Pkor10G02306.1* sequences. The presence of functional defects in *IAA16* led to a decreased response to auxin and abscisic acid, ultimately inhibiting plant growth [71]. This study discovered that the promoter region of *Pkor07G00704.1* contains a significant number of ABA-responsive elements. It suggests that *Pkor10G02306.1* may play a role in regulating ABA-mediated plant growth processes by interacting with *Pkor07G00704.1*. Phylogenetic analysis revealed that *ARF7* and *IAA14* are part of the same subfamily and share the highest homology with *Pkor01G00962.1* and *Pkor10G02306.1*, respectively. *ARF7* and *IAA14* play a negative role in regulating the expression of chlorophyll biosynthesis genes and impede chloroplast development, which ultimately results in a reduction in photosynthetic activity in plants [72]. The analysis of cis-acting elements revealed that the promoter region of *Pkor01G00962.1* contains numerous light-responsive elements, suggesting that both *Pkor01G00962.1* and *Pkor10G02306.1* play a role in regulating auxin signaling and chlorophyll metabolism in plant development. In conclusion, *Pkor10G02306.1* interacts with *Pkor01G00962.1* and *Pkor07G00704.1* to regulate multiple phytohormone signaling pathways, thereby influencing plant growth and development processes.

### Analysis of gene expression characteristics of *PkorARF* during embryonic development

Embryonic development plays a crucial role in the growth and development process of Korean pine. The RNA-seq results reveal that the expression of *Pkor03G02515.1*, *Pkor10G00543.1*, *Pkor08G00952.1*, and *Pkor08G00968.1* in Group IIIb is significantly induced throughout all stages of embryonic development (Fig. 6). It is worth noting that *AtARF1*/*2*, a member of the Group IIIb subfamily, has been previously reported to promote vesicle transport from the endoplasmic reticulum to the golgi apparatus and regulate cell proliferation and elongation [73]. The genes *Pkor03G02515.1*, *Pkor10G00543.1*, *Pkor08G00952.1*, and *Pkor08G00968.1* likely have significant roles in regulating the process of embryonic development. On the other hand, *AtARF10*, *AtARF16*, and *AtARF17* act as negative regulators during seed germination [74], *Pkor03G00080.1* and *Pkor07G02104.1*, which belong to the Group I subfamily along with *AtARF10*/*16*/*17*, may also function as negative regulators during embryonic development (Fig. 6). *AtARF2*, *AtARF6*, and *ARF8* are involved in the maturation process of floral organs [15, 75]. While *AtARF5* is responsible for the development of radicle initiation to root morphogenesis [76]. Similarly, RT-qPCR results indicate that *Pkor08G00968.1*, *Pkor07G00704.1*, and *Pkor03G00307.1* have the closest genetic relationship with *AtARF2*, *AtARF6*/*8*, and *AtARF5*, respectively. Furthermore, their expression is up-regulated throughout the embryonic development of Korean pine (Fig. 6), suggesting their potential role in embryonic development. Interestingly, Pkor01G00962.1, which forms the core of the protein interactions network, and Pkor07G00704.1 exhibited distinct expression patterns during embryonic development. *Pkor01G00962.1* demonstrated an initial up-regulation followed by down-regulation throughout embryonic development, whereas *Pkor07G00704.1* consistently displayed increased expression during the embryonic development of Korean pine (Fig. 6). These results further confirm that *Pkor01G00962.1* and *Pkor07G00704.1*, which are the core genes of the PkorARFs family, play significant roles in regulating the early embryonic development of plants. Therefore, conducting in-depth research on *PkorARF* members of Korean pine through genetic engineering technology in the future will also serve as a reference for exploring the biological functions of ARF genes in woody plants.

## Conclusions

As a key factor in the auxin signaling pathway, *ARF* plays an essential role in plant growth and development. In this study, genome-wide analysis of *ARF* in Korean Pine was performed for the first time. The study identified a total of 13 *PkorARF* genes, which were found to be unevenly distributed across six chromosomes of Korean Pine. Phylogenetic analyses revealed that these 13 *PkorARF* genes belong to four distinct subfamilies, with each subfamily exhibiting similar gene structures and conserved motifs. The promoter region of the *PkorARF* gene contains a large number of hormone-responsive elements, indicating the important role of *PkorARF* genes in mediating plant hormone signal transduction pathways. The results of RT-qPCR showed significant induction of *Pkor01G00962.1* and *Pkor07G00704.1*, which are core genes of *PkorARF*, during early embryonic development. These findings provide new insights into the functional analysis of *PkorARF* genes in growth and development, as well as the enhancement of Korean Pine germplasm resources.

### Electronic supplementary material

Below is the link to the electronic supplementary material.

Supplementary Material 1

Supplementary Material 2

Supplementary Material 3

Supplementary Material 4

Supplementary Material 5

Supplementary Material 6

Supplementary Material 7

Supplementary Material 8

Supplementary Material 9

Supplementary Material 10

Supplementary Material 11

## Data Availability

All websites used in this study are included in Supplementary Table 8. The datasets supporting the conclusions of this article are included within the article and its supplementary files.
