# Supplementary material for: Genome-wide identification and expression analysis of ARF gene family in embryonic development of Korean pine (Pinus koraiensis)
Source: BMC Plant Biol. 2024 Apr 10;24:267. doi: 10.1186/s12870-024-04827-w (PMC11005186; doi:10.1186/s12870-024-04827-w)
Supplement: Supplementary file 1 — Supplementary Material 1 [file 12870_2024_4827_MOESM1_ESM.docx]

**Supplementary Fig. 1 Tertiary structure of PkorARF protein**

The protein sequences of PkorARF members are aligned with the SWISS-MODEL website for protein homology modeling. The blue line in the model represents the part that is perfectly aligned with the SWISS-MODEL database, and the red line represents the prediction part
